# Tilden’s Extracts

**Published:** 1850-11

**Authors:** 


### ARTICLE VI.

### TILDEN'S EXTRACTS.

We call the attention of Physicians to the advertisement of Messrs. Tilden & Co., in the present number of the Journal. Much attention has been recently devoted to the procuring of reliable drugs. Amongst those who have conferred a favor on the profession in this particular, Messrs. Tilden & Co. hold a prominent position. Many physicians are little in the habit of prescribing extracts, for the reason that many of the articles of this class kept in the shops, are totally inert. It gives us pleasure to say that the Extracts of Messrs. Tilden & Co. have been thoroughly tested and found to be of most excellent quality. The manner of their preparation is such as to preserve the peculiar properties of the article from which they are manufactured, while they are put up in the style best adapted to preserve them from deterioration. Specimens may be seen at the office of the editors; and the extracts are on sale at the Drug stores of this city. M.

